# Pharmacological inhibition of ataxia-telangiectasia mutated exacerbates acute kidney injury by activating p53 signaling in mice

**DOI:** 10.1038/s41598-020-61456-7

**Published:** 2020-03-10

**Authors:** Masahiro Uehara, Tetsuro Kusaba, Tomoharu Ida, Kunihiro Nakai, Tomohiro Nakata, Aya Tomita, Noriko Watanabe-Uehara, Kisho Ikeda, Takashi Kitani, Noriyuki Yamashita, Yuhei Kirita, Satoaki Matoba, Benjamin D. Humphreys, Keiichi Tamagaki

**Affiliations:** 1 Department of Nephrology, Kyoto Prefectural University of Medicine, Kyoto, Japan; 2 Department of Cardiovascular Medicine, Graduate School of Medical Science, Kyoto Prefectural University of Medicine, Kyoto, Japan; 3 Division of Nephrology, Washington University School of Medicine in St. Louis, St. Louis, USA

**Keywords:** Acute kidney injury, Renal fibrosis

## Abstract

The DNA damage response after kidney injury induces cell cycle arrest in renal tubular epithelial cells, resulting in the secretion of pro-fibrotic cytokines, thereby promoting interstitial fibrosis in a paracrine manner. Phosphorylation of ataxia-telangiectasia mutated (ATM) is the initial step in the DNA damage response and subsequent cell cycle arrest; however, the effects of ATM inhibition on the injured kidney have not been explored. Pharmacological ATM inhibition by KU55933 in cisplatin-treated mice did not ameliorate, but instead exacerbated cisplatin-induced DNA damage and tubular injury, thereby increasing mortality. Analysis of isolated tubular epithelia by FACS from bigenic SLC34a1-CreERt2; R26tdTomato proximal tubular-specific reporter mice revealed that KU55933 upregulated p53 and subsequent pro-apoptotic signaling in tubular epithelia of cisplatin-treated mice, leading to marked mitochondrial injury and apoptosis. In addition, KU55933 attenuated several DNA repair processes after cisplatin treatment, including single-strand DNA repair and Fanconi anemia pathways, suggesting that DNA repair after dual treatment of cisplatin and KU55933 was not sufficient to prevent the cisplatin-induced tubular injury. Our study suggested that ATM inhibition does not increase DNA repair after cisplatin-induced DNA damage and exacerbates tubular injury through the upregulation of p53-dependent pro-apoptotic signaling. Acute kidney injury must be carefully monitored when ATM inhibitors become available in clinical practice in the future.

## Introduction

Society is aging worldwide, and the number of patients with end-stage organ failure involving the heart, lung, and kidney, and the costs of treating these diseases are increasing^1^. Recent epidemiological investigations demonstrated that a past history of acute kidney injury (AKI) is a strong determinant of the future incidence of CKD^2–4^. Maladaptive tubular repair after AKI is one of the underlying mechanisms for this process, eventually leading to tissue fibrosis and the subsequent loss of kidney function^5,6^.

Numerous insults, such as anticancer drugs and hemodynamic abnormalities, are known causes of AKI^7^. Cisplatin is a platinum-based anticancer drug that is widely used to treat many types of cancer, but the main dose-limiting side effect of cisplatin is nephrotoxicity^8,9^. Circulating cisplatin is transported into proximal tubular epithelia through the organic cation transporter 2 (OCT2), which is highly expressed in the basolateral membrane^10^. The accumulated cisplatin directly binds to DNA, which inhibits transcription and DNA replication, thereby inducing cell death^11,12^. For cell survival after injury, complex signaling pathways, known as the DNA damage response (DDR), are activated in the injured cells, and the detection and repair of DNA damage by transient cell cycle arrest are orchestrated to ensure the maintenance of genomic stability or integrity. In DDR, ataxia-telangiectasia mutated (ATM) and ataxia-telangiectasia and Rad3-related (ATR), which are members of the phosphatidylinositol 3 kinase-related kinase (PIKK) family, play central roles in the activation of cell cycle arrest and cell death in the case of severe DNA damage.

Regarding the impact of cell cycle arrest on the development of CKD after injury, the downstream signaling of ATM, such as p53 in the injured tubular epithelia, is controversial. Sustained G2/M arrested tubular epithelia after kidney injury *in vivo* resulted in kidney fibrosis via upregulation of the production of profibrotic cytokines like TGFβ and CTGF^13,14^. ATM inhibition *in vitro* inhibited cell cycle arrest after aristrochiac acid treatment and ameliorated the profibrotic gene upregulation. In addition, p53, a major downstream molecule of ATM, plays an essential role in apoptosis induction after injury, and the inhibition of p53 ameliorated kidney injury *in vivo*^13,15–19^. In contrast, Ma *et al*. reported that p53 inhibition exacerbated the tubular apoptosis induced by ischemia reperfusion injury^20^.

ATM inhibitors are emerging as candidate cancer therapeutics based on their effects on the increased hypersensitivity to irradiation of ATM-deficient cells^21–24^. In pre-clinical trials, transient inhibition of ATM led to radiosensitization or chemosensitization *in vivo* and *in vitro*^25–27^, thus it is possible that ATM inhibitors strengthen the effects of anti-cancer drugs or irradiation. For other disease conditions, genetic ATM deletion ameliorated doxorubicin-induced cardiotoxicity^28^ and pressure overload-induced heart failure^29^.

Considering these previous studies, whether inhibition of ATM and its downstream signaling exert renoprotective effects after injury is unclear. To address this controversy, we administered KU55933, a selective ATM inhibitor^27^, to mice with cisplatin-induced AKI. We found that marked tubular injury developed after combination treatment with cisplatin and ATM inhibitor, leading to a higher mortality rate. Analysis of isolated proximal tubular epithelia demonstrated p53 upregulation despite ATM inhibition during injury. These results suggest that the nephrotoxicity of cisplatin is exacerbated by ATM inhibition, and AKI must be carefully monitored when these therapies become available in clinical practice in the future.

## Results

### ATM inhibition increases the mortality of cisplatin-induced nephrotoxicity

**Figure 1:**
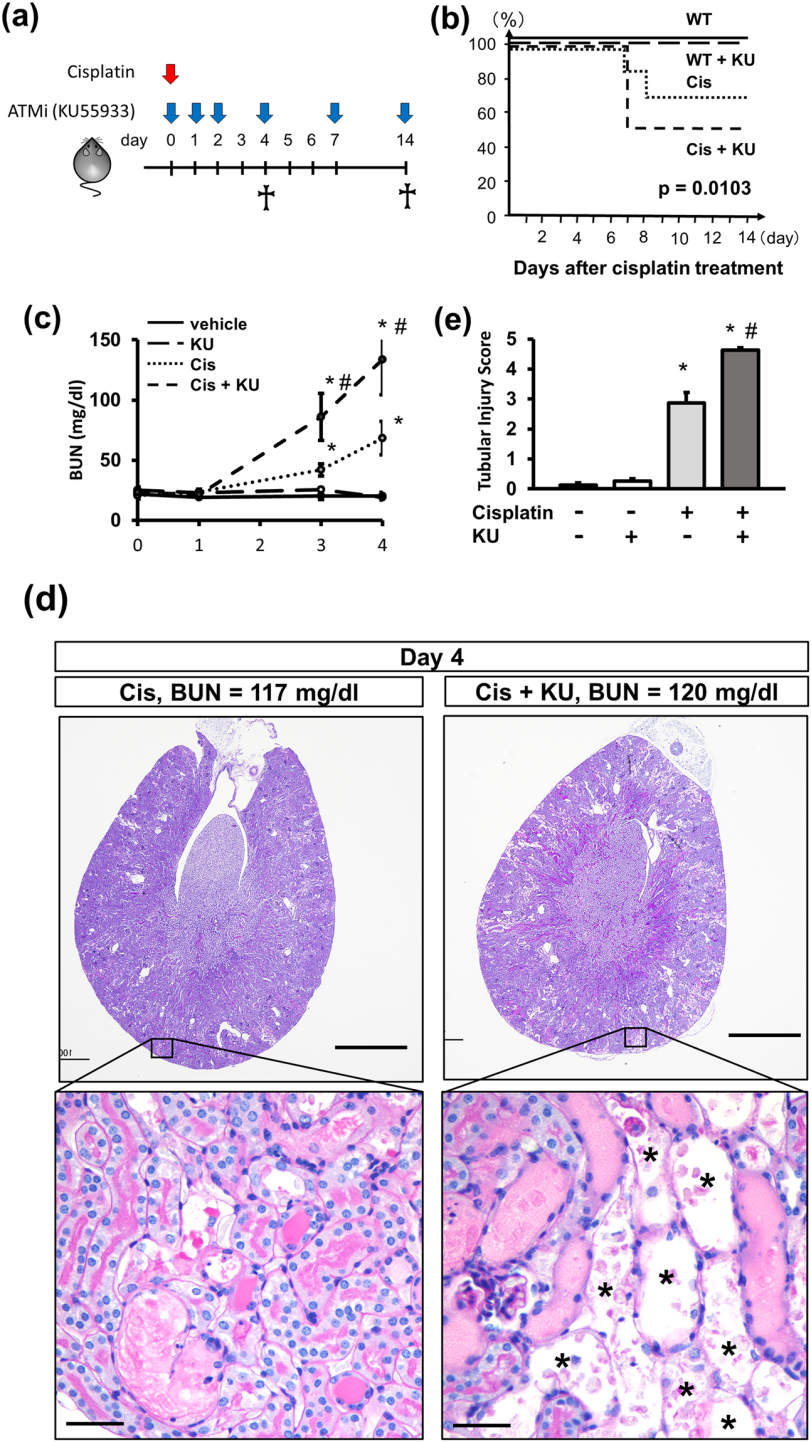
ATM inhibition increases the mortality rate and exacerbates tubular injury in cisplatin nephropathy. (**a**) Experimental scheme. Mice received intraperitoneal injections of cisplatin (15 mg/kg), KU55933 (5 mg/kg), or vehicle as indicated. (**b**) Kaplan-Meier curve for animal survival. KU55933 increased the mortality of cisplatin nephropathy. p = 0.0103 by the log rank test. n = 10 per group. (**c**) BUN increase in cisplatin nephropathy was accelerated by KU55933. n = 4–5 per group. (**d**) PAS staining of kidney sections (4 days after treatment) and (**e**) quantification of tubular injury score. Asterisks indicate the denuded tubular basement membrane. For all groups, data are means ± SEM, *p < 0.05 vs control, ^#^p < 0.05 vs cisplatin, Bar = 1 mm in low-power field pictures and = 50 μm in high-power field pictures in (**d**).

In order to investigate whether ATM inhibition can ameliorate kidney fibrosis at the chronic phase of cisplatin nephropathy, we injected KU55933 into mice that received cisplatin at the indicated time points (Fig. 1a). Co-administration of KU55933 and cisplatin significantly increased mortality (Fig. 1b). The BUN was significantly increased in the cisplatin-injected mice, and this increase was marked in the mice that received KU55933 and cisplatin (Fig. 1c). The kidneys of these mice exhibited extensive tubular injury characterized by detachment of tubular epithelia from the basement membrane and tubular cast formation (Fig. 1d,e), whereas KU55933 treatment alone did not induce morphological changes in the kidney (Supplementary Fig S1). Of note, numerous tubules with denuded tubular basement membrane where the tubular epithelia had completely detached were observed in the kidneys of mice that received KU55933 and cisplatin.

### ATM inhibition accelerates cisplatin-induced tubular injury

**Figure 2:**
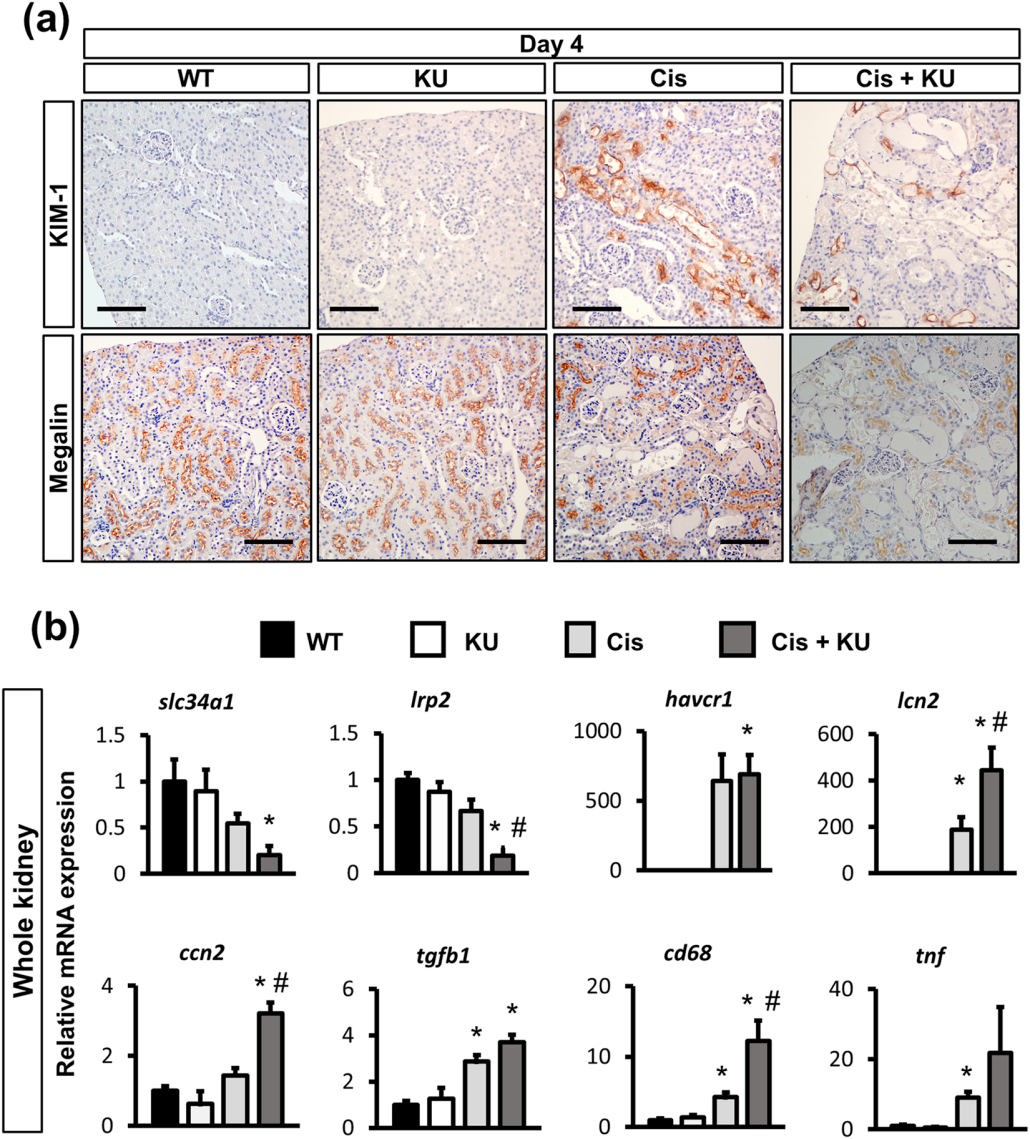
ATM inhibition upregulated proinflammatory and profibrotic signaling in cisplatin nephropathy. (**a**) Immunostaining of kidney sections for Kim-1 and megalin (4 days after treatment). (**b**) qPCR of RNA from whole kidneys for the representative markers of mature tubules (slc34a1 and megalin), of tubular injury (kim-1 and ngal), profibrotic cytokines (tgfb and ctgf), and inflammation (cd68 and tnfa). For all groups, data are means ± SEM, *p < 0.05 vs control, ^#^p < 0.05 vs cisplatin, Bar = 100 μm in (**a**).

To investigate the detailed morphological phenotypes, we assessed the expression of kim-1, a marker of tubular injury, and megalin, a marker of mature tubular epithelia. Immunostaining for kim-1 demonstrated a large number of positive tubules in the kidney of the mice that received cisplatin alone, whereas staining was much weaker in those that received KU55933 and cisplatin (Fig. 2a). In contrast, immunostaining for megalin was weak in the kidneys of the mice that received cisplatin with or without KU55933 (Fig. 2a). Of note, due to the complete detachment of tubular epithelia, tubules with denuded tubular basement membrane were negative for both megalin and kim-1, reflecting severe tubular injury (Supplementary Fig S2).

qPCR of whole kidney RNA confirmed the decreased expression of the mature tubular epithelia markers *slc34a1* and *lrp2* (encoding megalin) in cisplatin-injected mice, which was significantly lower in the mice that received KU55933 and cisplatin. *Havcr1* (encoding Kim-1) expression was upregulated in cisplatin-treated mice, and there was no difference between the mice with or without co-administration of KU55933. However, upregulation of another tubular injury marker, *lcn2* (encoding Ngal), was marked in the kidneys of the mice that received KU55933 and cisplatin. Profibrotic cytokines, *ccn2* and *tgfb1*, and inflammation markers, *cd68* and *tnf*, were upregulated in cisplatin-treated mice, and this upregulation was slightly higher in the mice that received KU55933 and cisplatin (Fig. 2b).

### ATM inhibition accelerates DNA damage in tubular epithelia

**Figure 3:**
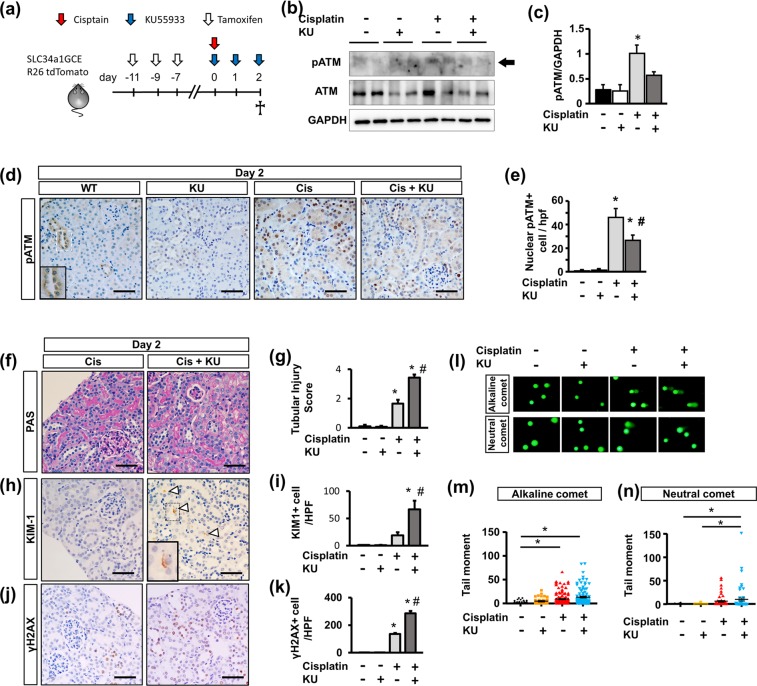
Acute effects of ATM inhibition on cisplatin nephropathy. (**a**) Experimental scheme. Bigenic mice (SLC34a1GCE x R26tdTomato) received intraperitoneal injections of cisplatin (15 mg/kg), KU55933 (5 mg/kg at each time point), Tamoxifen (3 mg/kg at each time point), or vehicle as indicated. (**b**). Western blot of protein lysates from whole kidneys for pATM, ATM, and GAPDH. Representative pictures of n = 2. Western blotting with n = 3 per group. (**c**) The optical density of pATM bands was normalized against that of GAPDH. (**d**) Immunostaining of kidney sections (2 days after treatment) for pATM and (**e**) quantification of nuclear pATM+ cells per low-power field picture (n = 4 per group). The small square in the WT image indicates the tubules with negative nuclear but high cytoplasmic staining. (**f**) PAS staining of kidney sections and (**g**) quantification of the tubular injury score. (**h**) Immunostaining of kidney sections for Kim-1 and (**i**) quantification of Kim-1+ cells per low-power field picture. (**j**) Immunostaining of kidney sections for γH2Ax and (**k**) quantification of γH2Ax + cells per low-power field picture. (**l**) Representative images of comet assay of kidney cells isolated from mice at 2 days after treatment and (**m,n**) quantitative analysis (M: alkaline comet: n = 100 each, N: neutral comet: n = 50 each). For all groups, data are means ± SEM, *p < 0.05 vs control, ^#^p < 0.05 vs cisplatin, Bar = 100 μm in (**d,f,h,j**).

As most of the severely injured tubular epithelia were lost due to substantial detachment from the tubular basement membrane 4 days after KU55933 and cisplatin treatment, we further analyzed the phenotypes at an earlier phase of 2 days after treatment. In addition, for tubular epithelia-specific analysis, we used bigenic mice with a proximal tubule-specific tamoxifen-inducible Cre (SLC34a1GCE) and the tdTomato reporter (R26tdTomato), in which proximal tubules were exclusively labeled by tdTomato after tamoxifen injection^30^. After labeling the proximal tubular epithelia by tamoxifen, the mice were used for experiments (Fig. 3a). Western blotting of whole kidney lysates revealed that KU55933 downregulated pATM expression in cisplatin-treated mouse kidneys (Fig. 3b,c). On immunohistochemistry of pATM, cisplatin increased the amount of nuclear pATM+ tubular epithelia, whereas it was significantly reduced by KU55933 (Fig. 3d,e). Regarding the specific expression of pATM in proximal tubular epithelia, immunostaining of pATM revealed that KU55933 significantly reduced the amount of pATM+ tdTomato+ tubular epithelia (Supplementary Fig S3a, S3b). Based on PAS staining, cisplatin-treated mice had tubular injury that was more severe in those that also received KU55933 (Fig. 3f,g). However, observation of detached tubular epithelia 4 days after treatment (Fig. 1d) was rare even in the mice that received both KU55933 and cisplatin. Based on immunostaining of KIM-1, the number of KIM-1+ cells was higher in mice that received KU55933 and cisplatin (Fig. 3h,i). To evaluate DNA damage as a consequence of cisplatin administration, we performed immunostaining of γH2AX. In both cisplatin-treated mice kidneys, a large number of γH2AX+ tubular epithelia were found, and this proportion was higher in the mice that received both KU55933 and cisplatin (Fig. 3j,k). In order to confirm the additional adverse effects of KU55933 on cisplatin-induced DNA damage and to elucidate the type of DNA damage in the kidney, we performed comet assay of isolated kidney cells from the mice 2 days after treatment. The comet tail moment in the alkaline condition (alkaline comet) reflects both DNA double stranded breaks (DSB) and single stranded breaks (SSB), whereas that in the neutral condition (neutral comet) reflects only DNA DSB^31^. We found that alkaline comet tail moments were increased in kidney cells from cisplatin treated mice, and were further increased in cells from both cisplatin- and KU55933-treated mice (Fig. 3l,m). Neutral comet tail moments were also increased in kidney cells from cisplatin-treated mice, and were further increased in those from both cisplatin- and KU55933-treated mice; however, the difference between the two groups was less for neutral comet tail moments (Fig. 3l,n). This suggests that DNA DSB and SSB occurred in the kidney after cisplatin administration, and that KU55933 accelerated the cisplatin-induced DNA damage, mainly DNA SSB.

Phosphorylation of ATR is another important responsive factor against DNA damage. In order to investigate the compensatory upregulation of ATR, immunostaining of pATR was performed. pATR+ cells were increased in cisplatin-treated mouse kidneys, and they were further increased in the mice that received both KU55933 and cisplatin (Supplementary Fig S4a, S4b).

### Tubular-specific phenotypes using FACS-sorted cells

**Figure 4:**
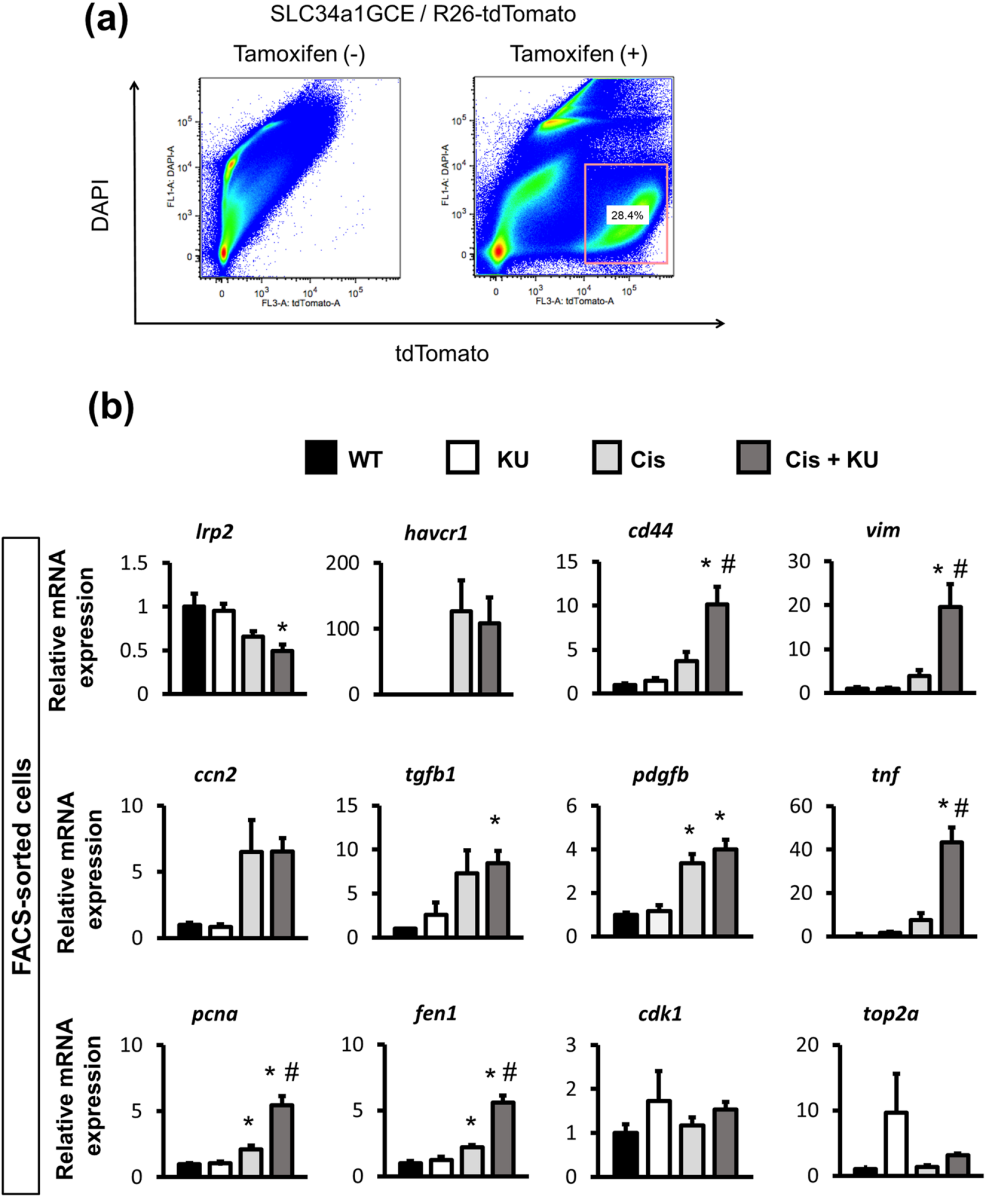
qPCR analysis of isolated proximal tubular epithelia from the mice that received cisplatin by FACS. (**a**) Isolation of tdTomato+ tubular epithelial cells using FACS as described in the experimental scheme in Fig. 3a. (**b**) qPCR of RNA from isolated tubular epithelia for the representative markers of mature tubules (*lrp2*), tubular injury (*havcr1*), dedifferentiation or mesenchymal markers (*cd44* and *vim*), profibrotic cytokines (*ccn2*, *tgfb1* and *pdgfb*), proinflammatory cytokines (*tnf*), and cell cycle (G1/S for *pcna* and *fen1*, G2/M for *cdk1* and *top2a*). For all groups, data are means ± SEM, *p < 0.05 vs control, ^#^p < 0.05 vs cisplatin.

In order to specifically analyze the phenotypes of proximal tubular epithelia, we collected the tdTomato+ tubular epithelia by fluorescence-activated cell sorting (FACS), and RNA and protein were extracted for further analysis (Fig. 4a). qPCR of isolated tubular epithelia demonstrated that RNA expression of *lrp2* and *slc34a1* was significantly reduced in the mice that received KU55933 and cisplatin (Fig. 4b). *Havcr1* expression was not affected by KU55933, but the expression of *vim* and *cd44*, which are mesenchymal markers, was significantly upregulated in the mice that received KU55933 and cisplatin (Fig. 4b), suggesting that the injured tubular epithelia underwent intratubular epithelial-mesenchymal transition, as observed on immunofluorescence images (Supplementary Fig S5). Regarding profibrotic and proinflammatory cytokines, *ccn2* and *tgfb1* expression did not differ between cisplatin with or without KU55933 (Fig. 4b). Regarding cell cycle markers, we evaluated the expression of regulatory molecules associated with the G1/S phase, including *pcna* and *fen1*, and the G2/M phase, including *cdk1* and *top2a*^32,33^. *Pcna* and *fen1* mRNA expression was increased in the mice that received KU55933 and cisplatin, whereas *cdk1* and *top2a* mRNA expression did not differ among all groups, suggesting that most cells arrested in the G1 phase^34^.

### ATM inhibition increased p53 expression in the tubular epithelia of cisplatin-treated mice

**Figure 5:**
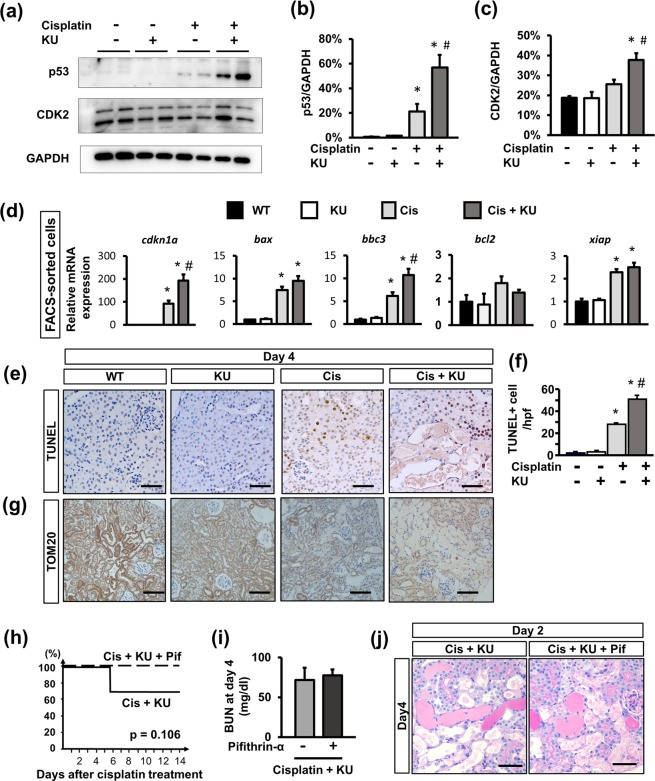
Activation of p53 signaling in proximal tubular epithelia in the cisplatin-treated mice with ATM inhibition. (**a**) Western blot of protein lysate from isolated tubular epithelia for p53, CDK2, and GAPDH. Representative pictures from n = 2. Western blotting with n = 4–6 in each group is shown. Optical density of (**b**) p53 and (**c**) CDK2 bands were normalized against those of GAPDH. The normalized density of the samples from the control mice was arbitrarily set to 1. (**d**) qPCR of RNA from isolated tubular epithelia for the downstream signaling of ATM and p53. (**e**) TUNEL staining of kidney sections (4 days after treatment) and (**f**) quantification of TUNEL + cells. (**g**) Immunostaining of kidney sections (4 days after treatment) for Tom20. (**h**) Kaplan-Meier curve for animal survival. Pifithrin-α slightly improved the mortality rate of cisplatin nephropathy after KU55933 administration. Log rank test. n = 8 in the pifithrin-α-treated group and n = 10 in the other group. (**i**) The BUN increase at 4 days after treatment did not differ between the two groups: n = 10 per group. (**j**) PAS staining of kidney sections (4 days after treatment). For all groups, data are means ± SEM, *p < 0.05 vs control, ^#^p < 0.05 vs cisplatin, Bar = 100 μm in (**e,j**) and = 50 μm in (**h**).

In order to explore the molecular mechanism in detail, we focused on p53 expression, which is major downstream signaling factor of ATM. Western blot analysis using isolated tubular epithelia by FACS demonstrated that cisplatin induced p53 and CDK2 expression, which was not attenuated but upregulated in the mice that received both KU55933 and cisplatin (Fig. 5a–c). Regarding further downstream signaling of p53, qPCR using isolated tdTomato+ tubular epithelia confirmed the upregulation of *cdkn1a* (encoding p21), and *bax*, and *bbc3* (encoding PUMA) in cisplatin-treated mice, which was slightly higher in the mice that also received KU55933 (Fig. 5d). Considering the upregulation of pro-apoptotic genes, we performed TUNEL staining to evaluate apoptotic cells. TUNEL+ cells were found in cisplatin-treated kidneys, and there were significantly more in the kidneys from mice treated with cisplatin and KU55933 (Fig. 5e,f). As previous studies found that cisplatin can induce mitochondrial injury through activation of the p53-PUMA axis^8^, we evaluated the expression of TOM20, a protein of the mitochondrial membrane. Immunostaining for TOM20 was weak in the kidneys from mice that received cisplatin, but it was even weaker in those of mice that received both KU55933 and cisplatin (Fig. 5g).

We further examined whether additional treatment with pifithrin-α, a selective p53 inhibitor, can prevent the acceleration of cisplatin nephropathy by ATM inhibition. Pifithrin-α slightly improved the mortality rate of cisplatin nephropathy with ATM inhibition (Fig. 5h), although it did not improve renal function or renal histology (Fig. 5i,j). These results suggested that pifithrin-α improved the repair process after severe renal injury by the co-administration of cisplatin and KU55933.

### ATM inhibition reduced DNA repair

**Figure 6:**
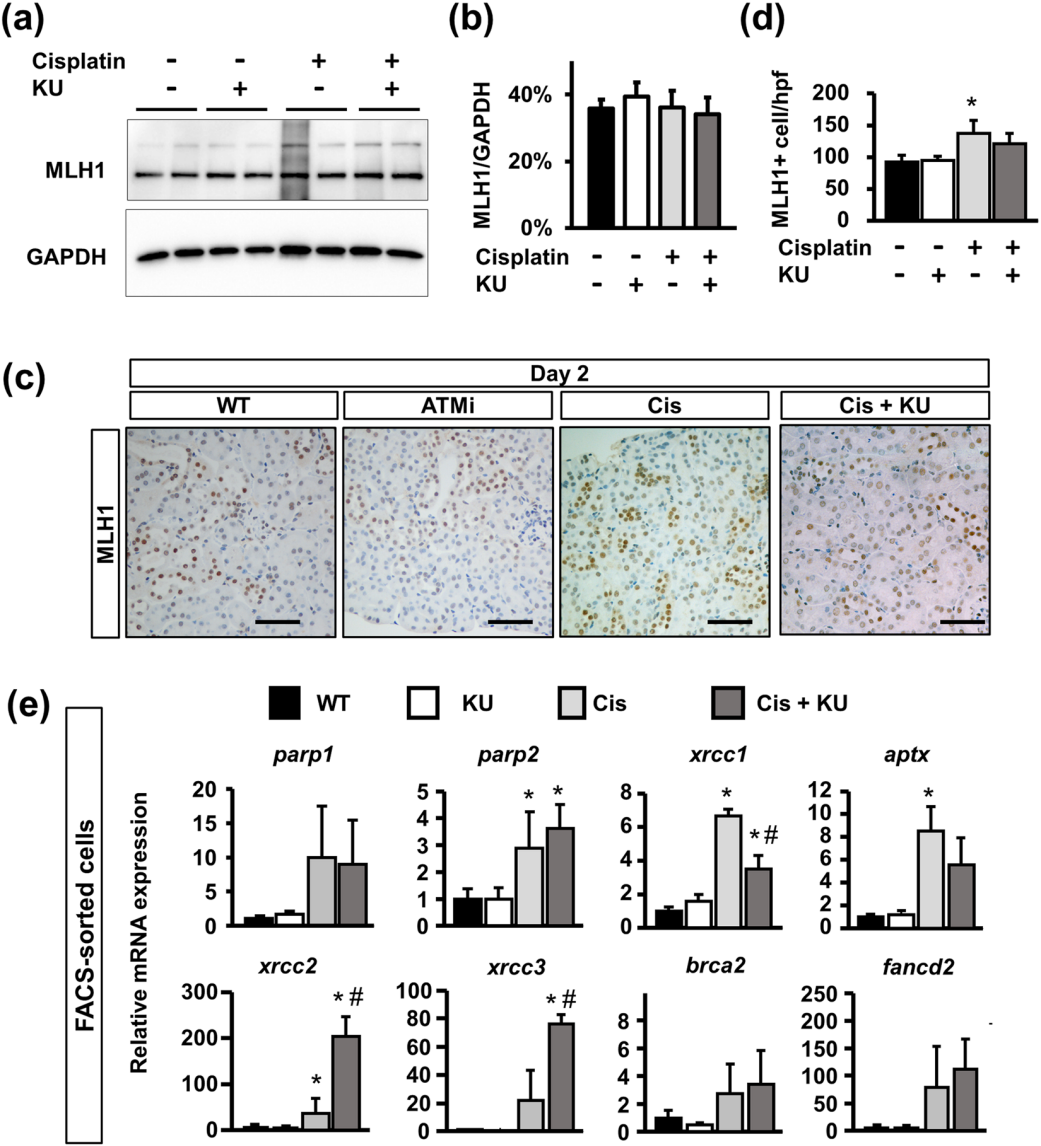
DNA repair in the tubular epithelia is reduced in mice that received cisplatin and the ATM inhibitor. (**a**). Western blot of protein lysates from isolated tubular epithelia for MLH1 and GAPDH. Representative pictures of n = 2. Western blotting with n = 4 per group. (**b**) Optical density of MLH1 bands was normalized against that of GAPDH. (**c**) Immunostaining of kidney sections (2 days after treatment) for Mlh1 and (**d**) quantification of nuclear MLH1 + cells per high-power field. (**e**) qPCR of RNA from isolated tubular epithelia for the genes related to DNA repair. For all groups, data are means ± SEM, *p < 0.05 vs control, ^#^p < 0.05 vs cisplatin, Bar = 50 μm in (**c**).

In response to cisplatin-induced DNA damage, several DNA repair pathways are activated and play essential roles in maintaining cell integrity and subsequent cell survival after injury. A previous report demonstrated the direct interaction of ATM and MutL homologue 1 (MLH1), which recognizes mismatched DNA and facilitates DNA mismatch repair (MMR)^35–37^, and was inhibited by KU55933^38^. Considering the negative regulatory effects on MMR of KU55933, we further analyzed its effects on the DNA repair process. Western blotting for MLH1 using isolated tdTomato+ tubular epithelia revealed that MLH1 expression was not affected by either cisplatin or the co-administration of cisplatin and KU55933 (Fig. 6a,b). On immunostaining analysis of MLH1, nuclear MLH1+ cells were found in all experimental groups (Fig. 6c). Quantification analysis revealed that the number of MLH1+ cells was larger in the mice that received cisplatin, whereas there were fewer in the mice that received both cisplatin and KU55933 (Fig. 6d), suggesting that ATM inhibition slightly reduced MMR after cisplatin-induced tubular injury.

Regarding the other DNA repair pathways, we analyzed DNA repair-related gene expression in FACS-sorted tubular epithelia; *parp1*, *parp2*, *xrcc1* and *aptx* for SSB repair, *xrcc2* and *xrcc3* for homologous recombination (HR), and *fancd2* and *bcra2* for the Fanconi anemia (FA) pathway (Fig. 6e). Although DNA damage was more marked in cisplatin- and KU55933-treated mice (Fig. 3l,m,n), expression of genes related to SSB repair and the FA pathway was similar to that in cisplatin-treated mice (Fig. 6e). Of note, *xrcc1* expression was slightly reduced in cisplatin- and KU55933-treated mice. However, the expression of *xrcc2* and *xrcc3* for HR exhibited a significant increase in cisplatin- and KU55933-treated mice. Therefore, KU55933 inhibits the adaptive DNA repair processes, especially MMR and FA pathways, after cisplatin treatment in tubular epithelia.

## Discussion

In order to elucidate the effects of ATM inhibition on injured proximal tubular epithelia, we administered KU55933 to mice with cisplatin-induced kidney injury and analyzed FACS-isolated proximal tubular epithelia, which were exclusively labeled by cre recombinase-mediated reporter expression. There were 4 major findings in our study. First, ATM was phosphorylated in tubular epithelial cells after kidney injury and ATM inhibition accelerated cisplatin nephrotoxicity, thereby increasing mortality. Second, ATM inhibition exacerbated cisplatin-induced DNA damage in proximal tubular epithelia. Third, regardless of ATM inhibition, p53 and its downstream signaling pathways were activated, thereby inducing significant tubular apoptosis. Fourth, multiple DNA repair processes against cisplatin-induced DNA damage were attenuated by ATM inhibition, resulting in maladaptive DNA repair after cisplatin treatment. Taken together, these results suggest that ATM plays a protective role against DNA damage in cisplatin-induced tubular epithelial injury.

Our study demonstrated that ATM inhibition exacerbates cisplatin-induced DNA damage, eventually leading to a higher mortality. Regarding DNA damage and the subsequent pro-apoptotic response, ATM and ATR are phosphorylated, which induces cell cycle arrest, and DNA repair is facilitated via their downstream targets. p53 is a major downstream molecule of ATM and is directly phosphorylated on serine 15 by pATM after DSB^39^. In our experiments, although immunostaining revealed a marked decrease in pATM expression in tubular epithelia by KU55933 treatment, the expression of tubular p53 and its downstream signaling paradoxically increased. p53 controls transcription-dependent and -independent mechanisms of downstream signaling. Low p53 expression results in the downregulation of cell cycle-related genes, such as p21, whereas higher expression induces pro-apoptotic responses such as PUMA expression^40^. PUMA, a strong regulator of the pro-apoptotic cascade through the mitochondrial pathway, accumulates in mitochondria, permeabilizes the mitochondrial membrane, and eventually induces mitochondrial injury^8^. Furthermore, cytoplasmic p53 itself accumulates in the mitochondrial matrix and induces mitochondrial injury^41,42^. These multifactorial pathways activated by ATM inhibition may have accelerated the mitochondrial injury and subsequent pro-apoptotic signaling observed in our study.

ATR, another central kinase involved in DDR, is activated by DNA SSB, whereas ATM is activated by DNA DSB^43^. Although these kinases are activated by different types of DNA damage, the downstream signaling responses partially overlap. Depletion of both ATR and ATM causes marked lethality following irradiation or administration of etoposide compared with the single depletion of ATR or ATM^44^. In addition, a recent study using mice with tubular epithelia specific deletion of ATR demonstrated that cisplatin-induced kidney injury was exacerbated and p53 was upregulated in mutant mice^45^, which is similar to our results of ATM inhibition. Considering these previous observations and our results of paradoxical p53 upregulation by KU55933, defects in ATR or ATM may be compensated by each other and upregulate p53. However, this compensatory effect was not sufficient to upregulate the DNA repair process, leading to the upregulation of pro-apoptotic signaling and exacerbated cisplatin-induced nephrotoxicity *in vivo*.

In response to non-severe cisplatin-induced DNA damage, DNA is repaired and cell death is prevented^46^. Cisplatin covalently binds to DNA bases and forms DNA adducts, blocking transcription and DNA synthesis^47^. There are two binding patterns between DNA and cisplatin, one is intrastrand adducts in which cisplatin binds to the same strand of DNA and the other is interstrand crosslink (ICL) in which cisplatin binds to the opposite strand of DNA. ICL occurs less frequently, less than 5% of total DNA platination, but it induces DSB, leading to severe DNA damage^47,48^. Several DNA repair processes function against cisplatin-induced DNA damage, including MMR, nucleotide excision repair (NER), HR, nonhomologous end joining (NHEJ), translesion synthesis (TLS), and FA pathways^49^. As ATM and ATR recognize the site of DNA damage, these directly or indirectly activate different DNA repair pathways.

Regarding the MMR after tubular injury, our study demonstrated that the combination of cisplatin and KU55933 slightly reduced nuclear MLH1 expression. Previous reports found that ATM is essential for stabilizing the complex of heterodimeric Mut, of which MLH1 is one of the major components^35^, and KU55933 decreased the association of these complexes^38^. In previous reports, the knockdown of MLH1 inhibited the DNA repair process^50^. Considering the significant expansion of DNA damage 2 days after cisplatin injection, reduced nuclear expression of MLH1 by KU55933 may inhibit the DNA repair process, thereby facilitating DNA damage and its downstream signaling.

Regarding the DNA repair process against ICLs, numerous pathways including NER, HR, and TLS, are required, and these are orchestrated by the FA pathway^51,52^. Of note, FANCD2, a central molecule of the FA pathway, is phosphorylated by ATM^53,54^. Although our comet study demonstrated that the frequency of DSB was not significantly different between cisplatin-treated mouse kidneys with or without KU55933, we comprehensively analyzed gene expression of molecules related to the FA and HR pathways. As a result, we found that cisplatin-induced activation of the FA pathway is attenuated by KU55933, HR pathways for *xrcc2* and *xrcc3* expression were significantly upregulated in response to the acceleration of DNA damage by KU55933. In the case of cisplatin-induced ICL, both FA and HR pathways synergistically accelerated DSB repair, but our results suggest that ATM mainly involves the FA pathway rather than the HR pathway.

Our study has several limitations. First, we found paradoxical upregulation of p53 in cisplatin- and KU55933-treated mouse kidney; however, the mechanism responsible for this upregulation is unclear. Considering the recent report demonstrating the similar upregulation of p53 in tubule-specific ATR knock-out mouse kidney^45^, and the compensatory interaction between ATR and ATM^44^, our results can be partially attributed to the upregulation of ATR, but future experiments are needed. Second, considering the paradoxical upregulation of p53 in cisplatin- and KU55933-treated mouse kidneys, we assumed that p53 was responsible for the exacerbation of kidney injury in our study. However, pifithrin-α did not ameliorate the severe renal histology of cisplatin- and KU55933-treated mouse kidneys, whereas it improved the mortality rate. Our results therefore cannot provide a reasonable explanation for this discrepancy, but another unknown pathway aside from p53-mediate apoptotic signaling may function in the development of severe renal histology. Third, unlike the cell-specific deletion of ATM, pharmacological inhibition of ATM may affect many cell types in the injured kidney. In a previous report, the cardioprotective effects of pharmacological ATM inhibition after doxorubicin treatment were attributed to its effects on interstitial fibroblasts rather than cardiomyocytes^28^. In contrast to such cardioprotective effects by pharmacological inhibition of ATM, KU55933 significantly exacerbated the cisplatin-induced nephrotoxicity; however, the effects of ATM inhibition on other cell types, including fibroblasts or other inflammatory cells, remain unclear.

In conclusion, we identified a central role for ATM in the balance of DNA damage and repair in cisplatin nephropathy. Our study suggests that should future anticancer therapies combine cisplatin with an ATM inhibitor, the risks of AKI may increase. For any drugs that can induce DNA damage in renal tubular epithelia, combination treatment with ATM inhibitors may accelerate tubular injury, thus careful monitoring of renal function or biomarkers should be required. In addition, as the mechanisms of the paradoxical upregulation of p53 under ATM inhibitor treatment have not been clarified, future experiments are required.

## Methods

### Animal experiments

We recently generated mice with the CreERT2 cassette in the SLC34a1 locus, which enables expression of the Cre recombinase in the proximal tubules after tamoxifen injection^30^. SLC34a1GCE mice were crossed with the R26tdTomato reporter mice in which tdTomato is expressed after Cre-mediated recombination of the floxed stop cassette to obtain bigenic offspring. For genetic labeling, tamoxifen (Sigma-Aldrich Co., LCC., St. Louis, MO) was dissolved in 3% (vol/vol) ethanol containing corn oil (Sigma Aldrich Co., LCC., St. Louis, MO) at a concentration of 10 mg/ml. Tamoxifen was injected intraperitoneally at the indicated dose either once or every other day.

The cisplatin injury model was induced by intraperitoneal injection of cisplatin (Nippon Kayaku, Tokyo, Japan) in PBS at a concentration of 15 mg/kg body weight into mice at the age of 9-12 weeks. Mice were euthanized 2, 4, or 14 days after the last cisplatin injection.

For ATM inhibition *in vivo*, 5 mg/kg of KU-55933 (ab120637, Abcam plc., Cambridge, UK) or vehicle (10% DMSO in saline) was intraperitoneally administered 30 minutes before, and 1, 2, 4, 7, and 14 days after cisplatin injection. For p53 inhibition *in vivo*, 2.2 mg/kg of pifithrin-α (ab120478, Abcam) was administered before cisplatin injection, and 2, 4, and 7 days after treatment. All experiments were approved by the Experimental Animals Committee, Kyoto Prefectural University of Medicine, and were performed in accordance with the institutional guidelines and Guidelines for Proper Conduct of Animal Experiments by the Science Council of Japan.

### Tissue preparation and histology

Mice were anesthetized and sacrificed, and kidneys were removed at the indicated time points. For frozen sections, kidneys were fixed with 4% paraformaldehyde (Wako Pure Chemical Industries, Ltd., Osaka, Japan) for 1 h on ice, incubated in 30% (vol/vol) sucrose in PBS at 4 °C overnight, and embedded in optimum cutting temperature compound (Sakura FineTek Japan co., Ltd., Tokyo, Japan) and cut into 7-μm sections.

For paraffin sections, the kidneys were fixed with 4% paraformaldehyde and embedded in paraffin by Applied Medical Research Laboratory (Osaka, Japan). Paraffin-embedded tissues were cut into 4-μm sections.

PAS and Masson’s trichrome staining was performed according to standard procedures. The kidney histology was examined on formalin sections stained with PAS and Masson’s trichrome. The degree of interstitial fibrosis or tubular injury was scored semi-quantitatively in five of 25 consecutive non-overlapping cortical fields of kidney sections stained with PAS and Masson’s trichrome under high magnification. Interstitial fibrosis was quantified using the following scores: 0, 0%; 1, 1–10%; 2, 11–25%; 3, 26–50%; 4, 51–75%; and 5, 76–100%. Tubular injury was judged by tubular atrophy, tubular dilation, protein casts, necrotic cells, and brush border loss^13,55^.

### Immunofluorescence analysis

Sections were rehydrated and permeabilized with 0.5% Triton X-100 in PBS for 5 min. Samples were blocked with 10% normal goat serum in PBS and incubated with primary antibodies, as listed in Supplementary Table 1. Samples were then incubated with secondary antibodies, anti-rabbit or -rat Alexa Fluor 488-conjugate (Thermo Fisher Scientific, Waltham, MA), for 1 h. When the mouse antibody was used as the primary antibody, we used the Zenon Alexa Fluor 488 Mouse IgG1 Labeling Kit (Z25002: Thermo Fisher Scientific, Waltham, MA) in order to avoid cross-reactivity of the secondary antibody against the resident mouse IgG in the tissue. Nuclear counterstaining was performed using DAPI or DRAQ5 (DR50050; BioStatus; 1:1000), followed by mounting in Prolong-Gold (Thermo Fisher Scientific). Images were obtained by confocal microscopy (FV1000; Olympus, Tokyo, Japan). pATM+ tubular epithelia among tdTomato+ cells were quantified from five of 25 consecutive non-overlapping cortical fields in each kidney under high magnification (n = 5). These five fields were randomly selected in a blinded manner.

### Immunohistochemistry and TUNEL assay

After deparaffinization, paraffin sections were placed in citrate-buffered solution (pH 6.0) and boiled for 5 minutes to retrieve antigens. Endogenous peroxidase was quenched with 3.0% hydrogen peroxide in methanol for 20 minutes. Samples were blocked with 3% BSA in PBS for 30 min at room temperature and incubated with primary antibodies (Supplementary Table 1). The sections were labeled with HRP-conjugated secondary antibodies, goat anti-rabbit (ab236469, abcam) or donkey anti-goat (sc-2020; Santa Cruz Biotechnology, Inc. Dallas, TX, USA; 1:500). Diaminobenzidine (DAB) chromogenic substrate (K3468, Agilent Technologies, Inc., Santa Clara, CA) was used for the color reaction, followed by counterstaining with hematoxylin. TUNEL staining for paraffin sections of the mouse kidney at 4 days after the treatments was performed using the ApopTag® Peroxidase *In Situ* Apoptosis Detection Kit (Merck S7100, Darmstadt, Germany) according to the manufacturer’s protocol. All sections were observed using the Eclipse E600 microscope (Nikon Corporation, Tokyo, Japan). pATM-, pATR-, γH2AX-, KIM-1-, MLH1-, and TUNEL-positive cells were quantified from five of 25 consecutive non-overlapping cortical fields in each kidney under high magnification (n = 5). These five fields were randomly selected in a blinded manner.

### Separation of tdTomato-Positive Proximal Tubular Epithelia using FACS

The kidney cortex was minced and a single-cell suspension was generated via research grade Liberase TL (5401020001:Sigma-Aldrich) and 60 units/ml of DNAse Inhibitor (D5025:Sigma-Aldrich) for 30 min at 37 °C.Cells were washed twice with PBS, filtered through 70- and 40-μm cell strainers, resuspended in PBS and 2%FBS with 1000:1 DAPI (1 mg/mL), and subjected to FACS using SH800 (SONY). Dead cells (DAPI+) were excluded during FACS. TdTomato+ DAPI- cells were collected in DMEM and 10% FBS. RNA and protein were extracted using NucleoSpin^®^ TriPrep (TAKARA Bio Inc, Shiga Japan).

### RNA extraction and real-time quantitative PCR

Total RNA was isolated from the kidneys or FACS-sorted cells using TRIzol (Life Technologies, Inc., Carlsbad, CA) and Direct-zol^TM^ RNA MiniPrep (Zymo Research Corporation., Irvine, CA) according to the manufacturer’s protocol. Subsequently, complementary DNA (cDNA) was generated using a Prime Script reverse transcription (RT) reagent kit (RR0471A Takara Bio Inc., Shiga, Japan), and real-time PCR was performed using KAPA SYBR Fast universal (KK4602: Kapa Biosystems, Wilmington, MA) and a Thermal Cycler Dice Real Time System (Takara Bio Inc., Shiga, Japan). All reactions were performed in duplicate. An initial denaturation step was performed for 10 min at 95 °C, and was followed by 45 cycles of amplification at 95 °C for 10 s, 62 °C for 10 s, and 72 °C for 30 s. Gene expression was quantified using GAPDH as an internal control. The primers used are listed in Supplementary Table 2.

### Western blot analysis

Total cell extracts were obtained using lysing buffer 17 (895943: R&D Systems, Inc. Minneapolis, MN, USA) for 5 min at room temperature. After sonication, proteins were denatured by heating for 5 min at 95 °C and separated by SDS-PAGE. Subsequently, proteins were transferred onto polyvinylidene difluoride (PVDF) membranes (Immobilon-P IPVH00010: Millipore, MA, USA). After blocking in 5% non-fat milk in TBS/0.1% Tween20 for 1 h at room temperature, the membrane was incubated with the corresponding primary antibody (Supplementary Table 1) overnight at 4 °C. After washing with TBS/0.1% Tween20, secondary peroxidase-conjugated anti-mouse or anti-rabbit antibodies were added (7076S, 7074S; Cell Signaling Technology, Boston, MA; 1:3000 at room temperature). Chemiluminescence was detected using an ECL select Western blot detection reagent (RPN2235: GE Healthcare UK Ltd, Amersham Place, England) or Clarity Max western ECL substrate (1705062: Bio-Rad Laboratories, Inc. Hercules, CA, USA). Signal intensities were evaluated using ImageJ software (National Institutes of Health, Bethesda, MD).

### Comet assay

The comet assay was performed using the Comet Assay Kit (abcam ab238544) according to the manufacturer’s protocol. In brief, mouse kidneys were removed and minced in a small amount of ice-cold PBS containing 20 mM EDTA. After removing the large pieces of tissue, the supernatant was passed through a 35-μm cell strainer. After centrifugation, the pellet was suspended at 1 × 10^5^ cells/ml in ice-cold PBS. Cell samples were mixed with comet agarose in a 1/10 ratio (v/v) and immediately transferred onto the slide glasses covered with comet agarose base layer. After incubating with pre-chilled lysis buffer, the slides were subjected to electrophoresis. Electrophoresis was performed in the Alkaline Electrophoresis Solution for the alkaline comet assay and in the TBE Electrophoresis Solution for the neutral comet assay. After electrophoresis, the slides were incubated with Vista Green DNA dye. Images were obtained by epifluorescence microscopy (IX71; Olympus, Tokyo, Japan) using the FITC filter. Ten pictures (5–15 cells per picture) were randomly taken, and the tail moment (tail length x tail % DNA/100) of 100 cells in the alkaline test and 50 cells in the neutral test per group was calculated using Comet Score analysis software (TriTek Corp.).

### Statistics

Results are expressed as the mean ± SEM. Each experiment was performed using at least three mice per group. Statistical analysis was performed by the unpaired t-test for comparison of two variables, and by analysis of variance and Dunnett’s *post hoc* test for comparison of multiple variables. Survival curves after cisplatin treatment were analyzed by the Kaplan-Meier method and comparison of the two groups was performed using the Log rank test. P-values < 0.05 were considered significant.

## Supplementary information

Supplementary figures and tables.

Author_List_Changes_Approval_form.
