# Supplementary material for: Pharmacological inhibition of ataxia-telangiectasia mutated exacerbates acute kidney injury by activating p53 signaling in mice
Source: Sci Rep. 2020 Mar 10;10:4441. doi: 10.1038/s41598-020-61456-7 (PMC7064514; doi:10.1038/s41598-020-61456-7)
Supplement: Supplementary file 1 — Supplementary figures and tables. [file 41598_2020_61456_MOESM1_ESM.docx]

**Supplementary Information**

**Pharmacological inhibition of ataxia-telangiectasia mutated exacerbates acute kidney injury by activating p53 signaling in mice**

Masahiro Uehara, Tetsuro Kusaba, Tomoharu Ida, Kunihiro Nakai, Tomohiro Nakata, Aya Tomita, Noriko Watanabe-Uehara, Kisho Ikeda, Takashi Kitani, Noriyuki Yamashita, Yuhei Kirita, Satoaki Matoba, Benjamin D. Humphreys and Keiichi Tamagaki

**2 supplementary tables and 6 supplementary figures**

**Supplementary Table 1**. The primary antibodies for Western blotting and immunostaining

| Protein | Source | Catalog # | Vendor |
| --- | --- | --- | --- |
| KIM-1 | Goat | AF1817 | R&D |
| Megalin | Goat | sc-16478 | SantaCruz |
| pATM | Mouse | ab36810 | abcam |
| pATM (HRP conjugated) | Mouse | sc-47739 HRP | SantaCruz |
| pATR | Rabbit | 2853S | CST |
| Vimentin | Rabbit | ab92547 | abcam |
| γH2AX | Rabbit | 2577S | CST |
| p53 | Mouse | 2524S | CST |
| Cleaved caspase3 | Rabbit | 9664S | CST |
| TOM20 | Rabbit | sc-11415 | SantaCruz |
| CDK2 | Rabbit | ab32147 | abcam |
| MLH1 | Rabbit | ab92312 | abcam |
| FANCD2 | Rabbit | Ab108928 | abcam |
| PDGFRβ | Mouse | NBP1-47232 | Novus |
| GAPDH (HRP-conjugate) | Mouse | ab105428 | abcam |

**Supplementary Table 2**. The primers for qPCR

| Gene | Forward | Reverse |
| --- | --- | --- |
| *slc34a1* | ACAAAACCCTACTGGGTGGA | CTCGCTGTAGGACATCAT |
| *lrp2* | AAAATGGAAACGGGGTGACTT | GGCTGCATACATTGGGTTTTCA |
| *havcr1* | AAACCAGAGATTCCCACACG | GTCGTGGGTCTTCCTGTAGC |
| *lcn2* | GCAGGTGGTACGTTGTGGG | CTCTTGTAGCTCATAGATGGTGC |
| *ccn2* | GGCCTCTTCTGCGATTTCG | GCAGCTTGACCCTTCTCGG |
| *tgfb1* | GAACCCCCATTGCTGTCCC | AGCCCTGTATTCCGTCTCCT |
| *tnf* | AAGGCTGCCCCGACTACG | AGGTTGACTTTCTCCTGGTATGAG |
| *cd68* | TTCTGCTGTGGAAATGCAAG | AGAGGGGCTGGTAGGTTGAT |
| *cd44* | TGAAACATGCAGGTATGGGT | GCTGAGGCATTGAAGCAATA |
| *vim* | GCTGCGAGAGAAATTGCAGGA | CCATTTCCGTTCAAGGTCAAG |
| *pdgfb* | TGCTGCACAGAGACTCCGTA | GATGAGCTTTCCAACTCGACTC |
| *pcna* | TTGCACGTATATGCCGAGACC | GGTGAACAGGCTCATTCATCTCT |
| *fen-1* | ACCAGTTCCTGATTGCTGTTC | TCATGCGGATGGTACGGTAGA |
| *cdk1* | AGGTACTTACGGTGTGGTGTAT | CTCGCTTTCAAGTCTGATCTTCT |
| *top2a* | TGCTCCGCCCAGATACCTAC | TGGGTCCCTTTGTTTGTTATCAG |
| *cdkn1a* | GTGGGTCTGACTCCAGCCC | CCTTCTCGTGAGACGCTTAC |
| *bax* | CCGGCGAATTGGAGATGAACT | CCAGCCCATGATGGTTCTGAT |
| *bbc3* | TGTGGAGGAGGAGGAGTGG | TGCTGCTCTTCTTGTCTCCG |
| *bcl-2* | GCTACCGTCGTGACTTCGC | CCCCACCGAACTCAAAGAAGG |
| *xiap* | AGTTGTCATGCGGCAATAGATAG | CTGTCAGGGGCAAAAGGATTT |
| *parp1* | **GTGACTTTTTAGCGGAGTACGC** | **CCAGCGGTCAATCATACCCAG** |
| *parp2* | **CAGCACGCAGGATGAAAGTAA** | **CTGGTAACCGGCCTTGATTTG** |
| *xrcc1* | **TCCACCCTCAAGAGACCCAAA** | **AGCGGAAGGGGTTCTGGAA** |
| *aptx* | **TGCTGGTTGGTGAGACAGGA** | **ACTCTGCTTTCAACTGTACTTGC** |
| *xrcc2* | **TACCACTTTGACATGCTACGGC** | **CACTGACTCGGTCTATCCAGTA** |
| *xrcc3* | **CGAATTACTGCTGCGGTTAAGA** | **CCCGAAGGTGTAGAGAGGCA** |
| *brca2* | **ATGCCCGTTGAATACAAAAGGA** | **ACCGTGGGGCTTATACTCAGA** |
| *fancd2* | **TAATGGCCTGGAGTCCTACAC** | **CTCTTGGAGTAAAATGTGCCCA** |
| *gapdh* | TGCGACTTCAACAGCAACTC | CTTGCTCAGTGTCCTTGCTG |

**Supplementary Figure 1**. PAS staining of vehicle- or KU55933-treated mouse kidneys. No significant histological changes were found. Bar = 50 μm


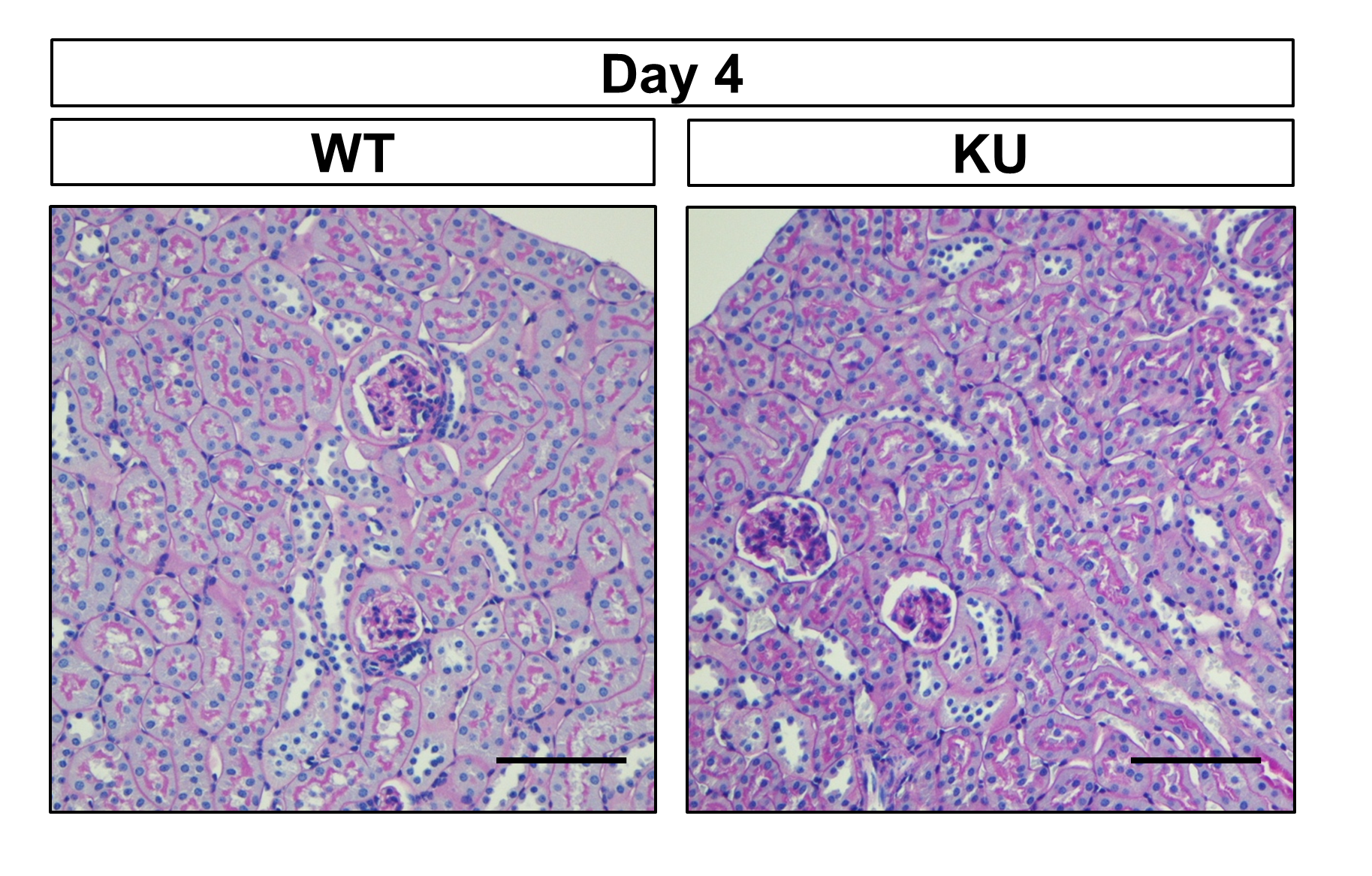


**Supplementary Figure 2**. Immunostaining of serial kidney sections (2 days after treatment) for kim-1 and megalin. Arrowheads indicate denuded tubules that are negative for both megalin and kim-1. Bar = 20 μm


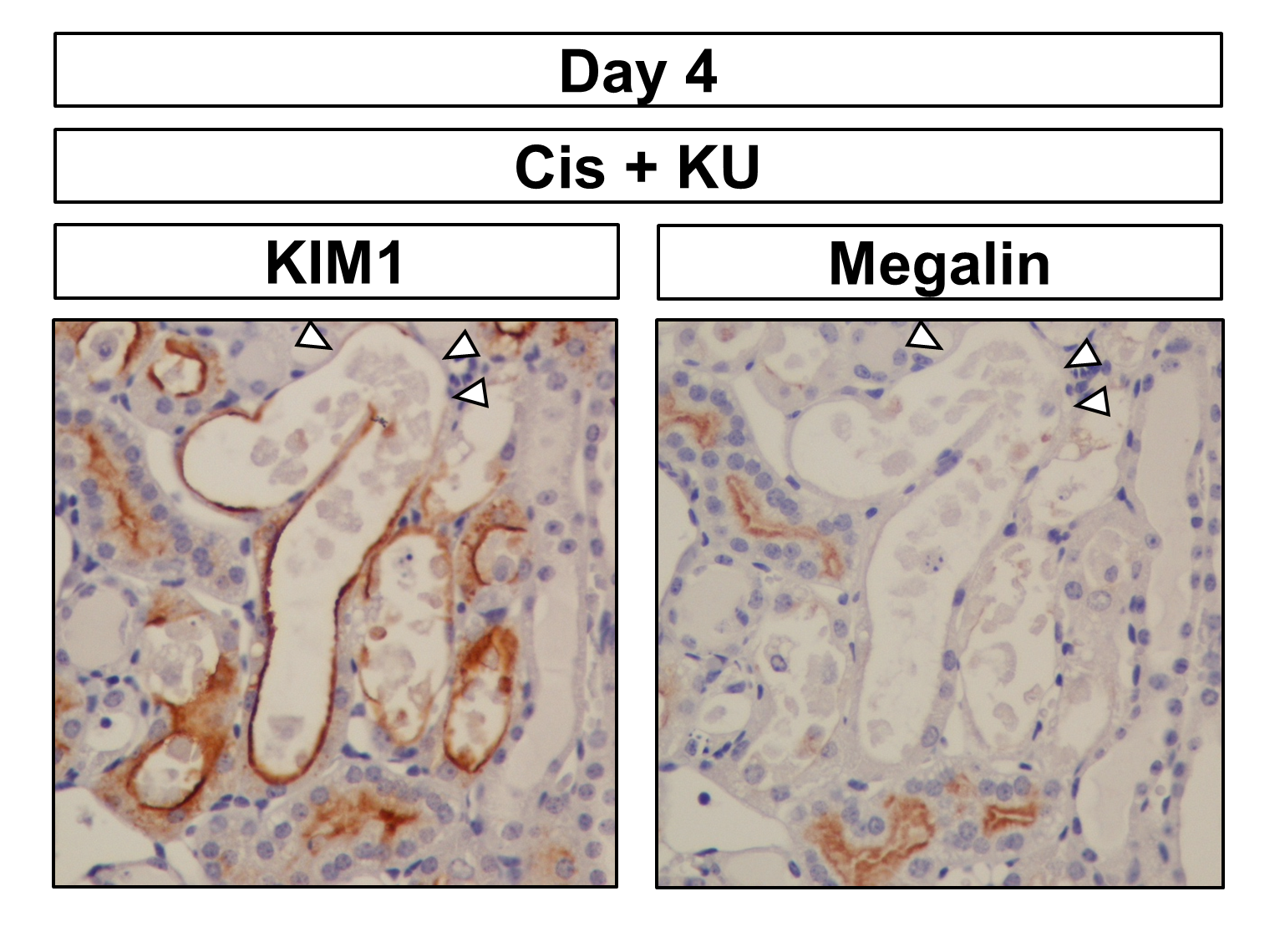


**Supplementary Figure 3**. (a) Immunofluorescence of pATM in kidney sections (2 days after treatment). Small arrowheads indicate pATM+ nuclei. (b) Quantification of nuclear pATM+ cells among tdTomato+ tubular epithelia. (n = 5 per group). For all groups, data are means ± SEM, * p < 0.05 vs control, # p < 0.05 vs cisplatin, Bar = 50 μm

**
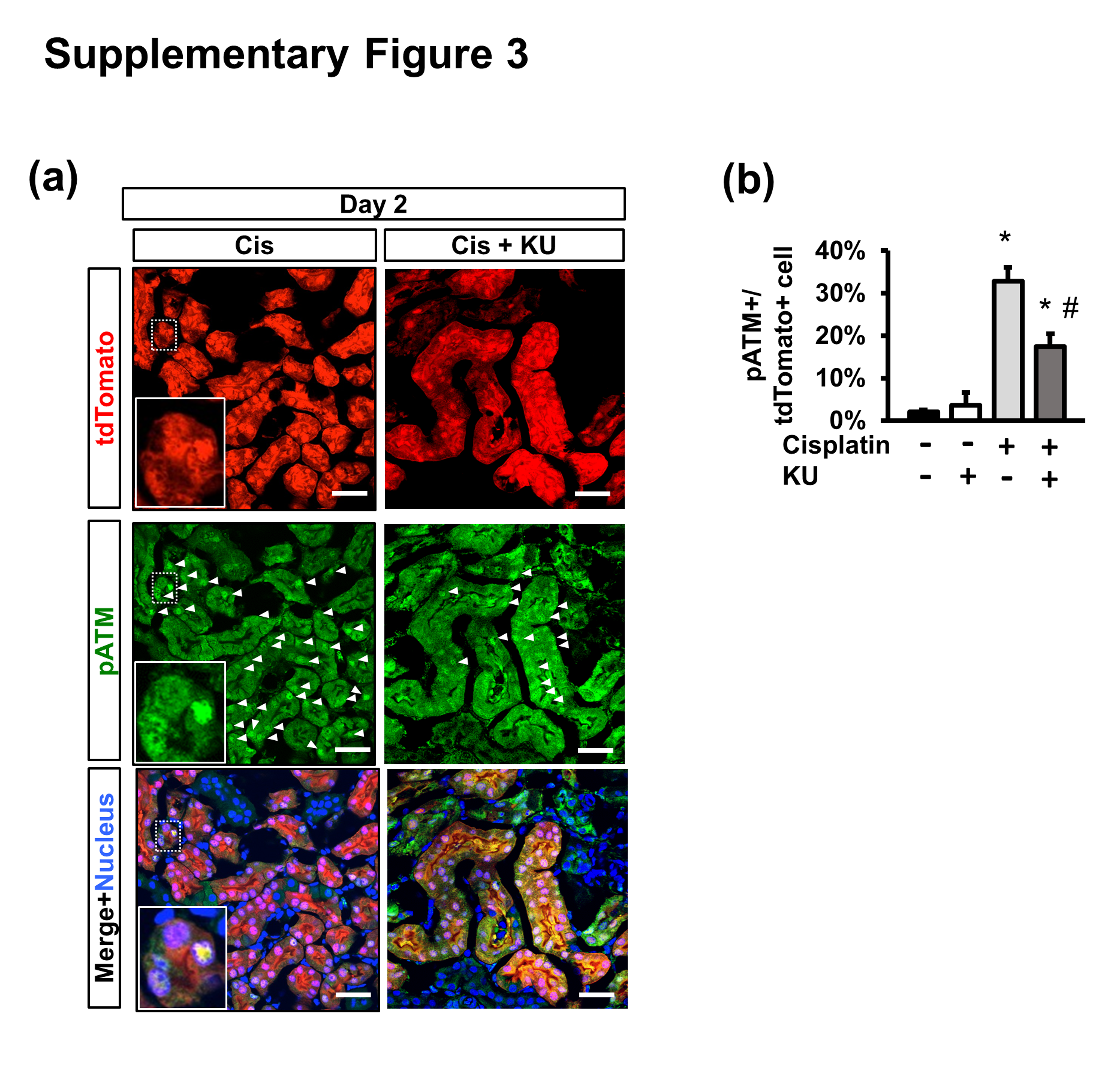
**

**Supplementary Figure 4**. (a) Immunohistochemistry of kidney sections (2 days after treatment) for pATR and (b) quantification of the number of pATR+ tubular epithelia. For all groups, data are means ± SEM, * p < 0.05 vs control, # p < 0.05 vs cisplatin, Bar = 50 μm


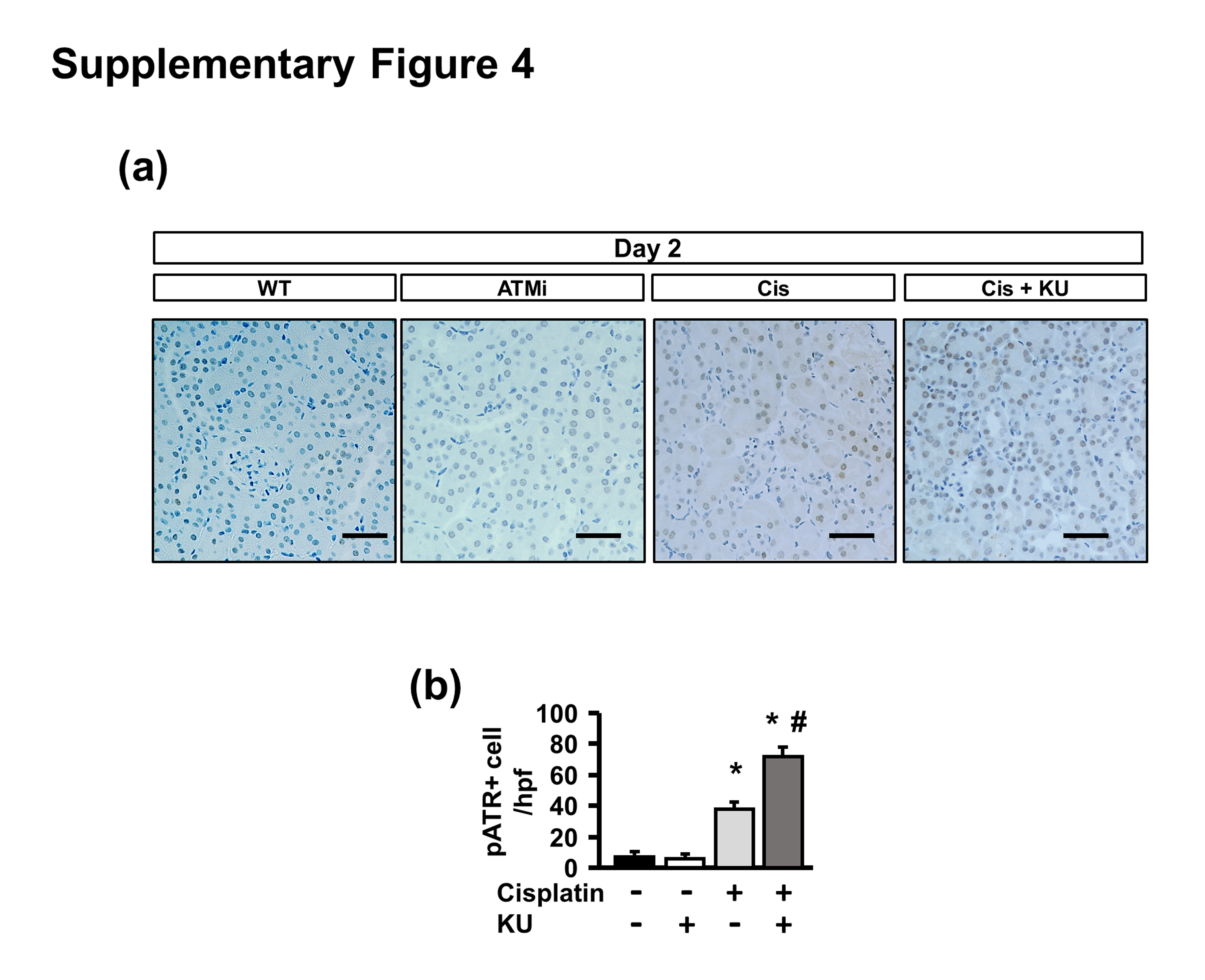


**Supplementary Figure 5**. Immunofluorescence of vimentin in kidney sections (2 days after treatment). Arrowheads indicate the tdTomato+ vimentin+ tubular epithelial cells undergoing intratubular EMT. A higher magnification image is shown in the small square. Bar = 50 μm


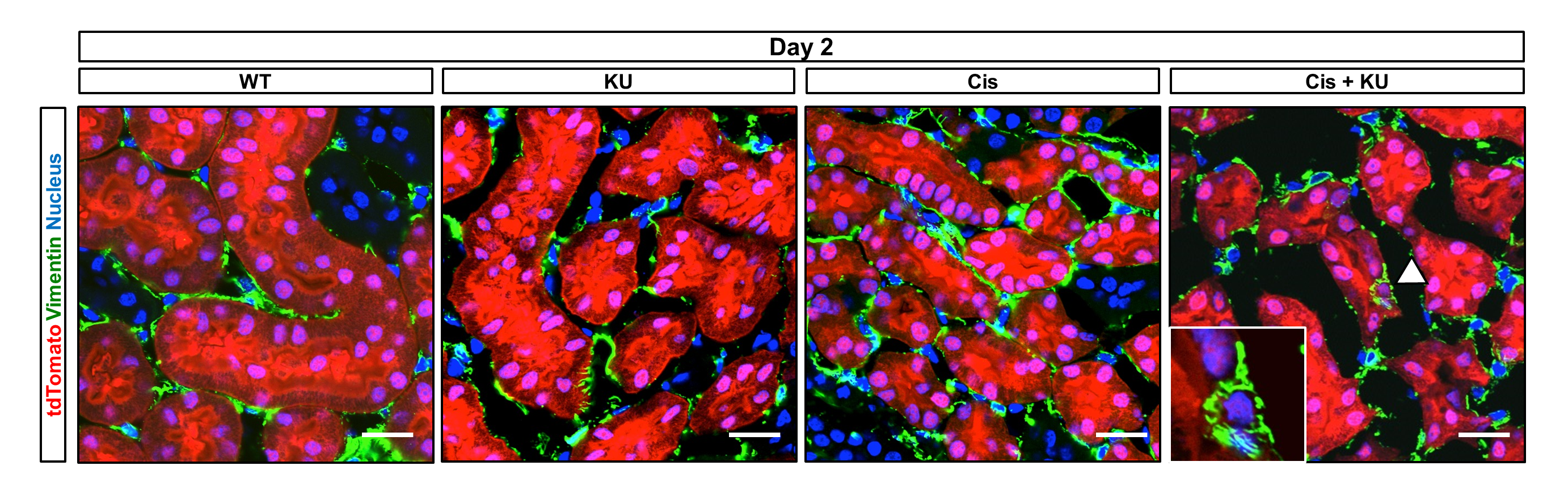


**Supplementary Figure 6**


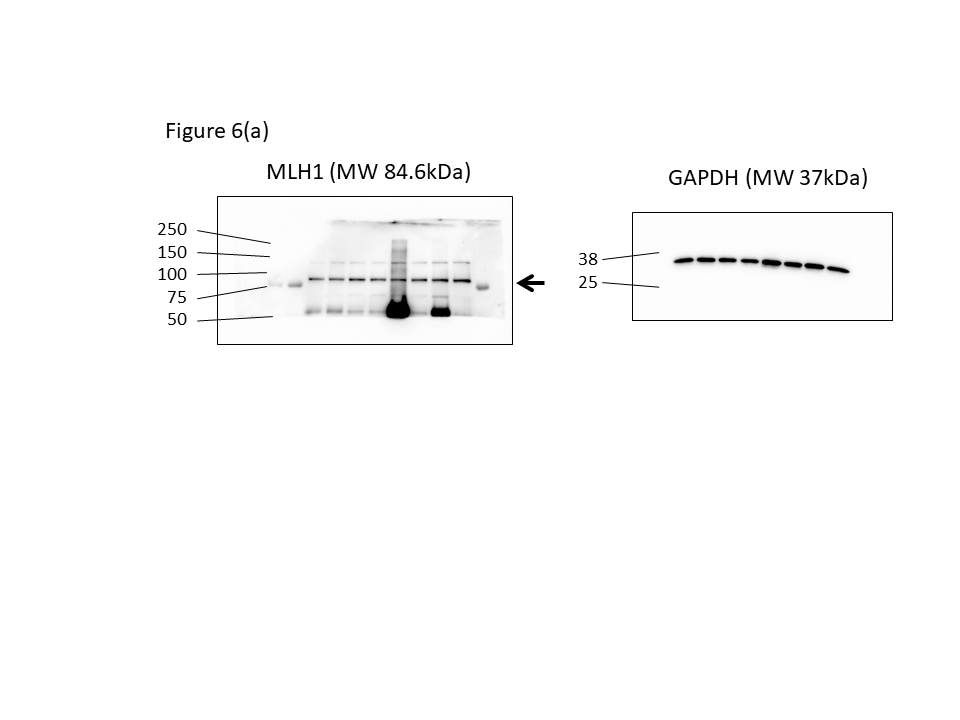
**
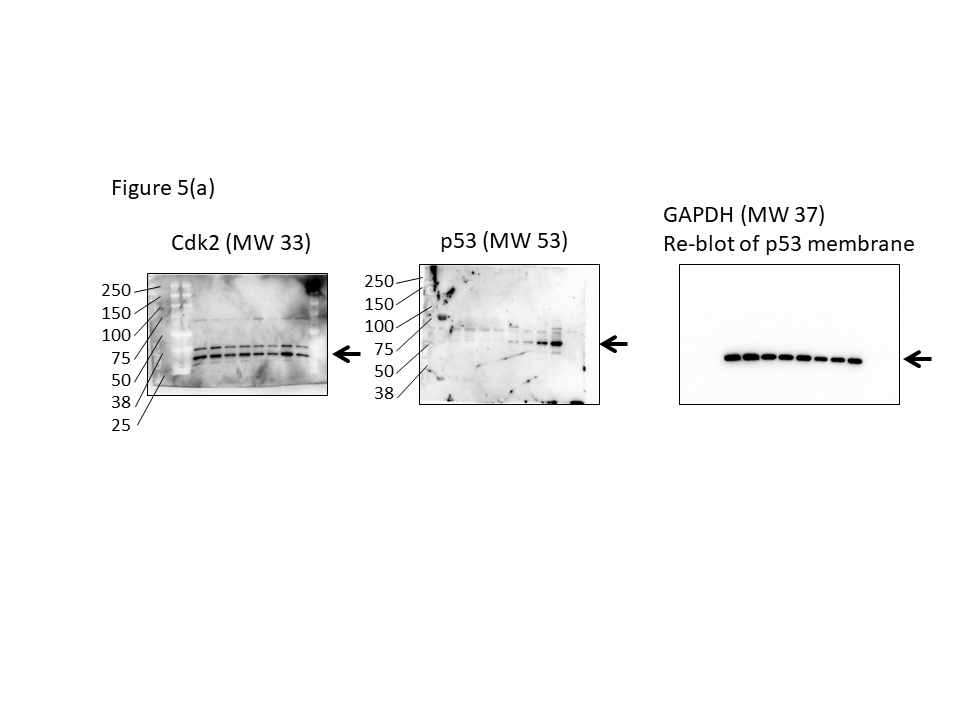

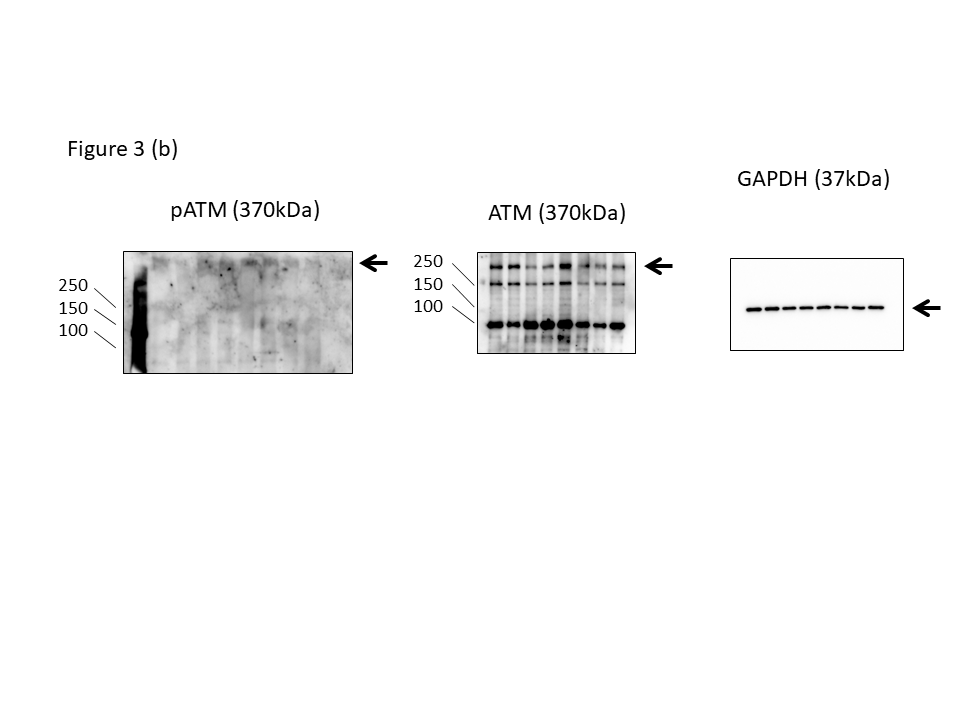
**Unprocessed Western blots for the figures
