# Supplementary material for: Pharmacological inhibition of ataxia-telangiectasia mutated exacerbates acute kidney injury by activating p53 signaling in mice
Source: Sci Rep. 2020 Mar 10;10:4441. doi: 10.1038/s41598-020-61456-7 (PMC7064514; doi:10.1038/s41598-020-61456-7)
Supplement: Supplementary file 2 — Author_List_Changes_Approval_form. [file 41598_2020_61456_MOESM2_ESM.pdf]

nature publishing group npg

In accordance to Nature Publishing Groups Authorship Policy we agree to change the authors of the manuscript as indicated below.

NAME OF JOURNAL: **Scientific Reports**

TITLE OF MANUSCRIPT: **Pharmacological inhibition of ataxia-telangiectasia mutated exacerbates acute kidney injury by activating p53 signaling in mice**

MANUSCRIPT NUMBER: **SREP-19-36439A**

CORRESPONDING AUTHORS NAME: **Tetsuro Kusaba**

PREVIOUS AUTHOR NAMES:
Masahiro Uehara, Tetsuro Kusaba, Tomoharu Ida, Tomohiro Nakata, Aya Tomita, Noriko Watanabe-Uehara, Kisho Ikeda, Takashi Kitani, Noriyuki Yamashita, Yuhei Kirita, Satoaki Matoba, Benjamin D. Humphreys, Keiichi Tamagaki

UPDATED AUTHOR NAMES:
Masahiro Uehara, Tetsuro Kusaba, Tomoharu Ida, Kunihiro Nakai, Tomohiro Nakata, Aya Tomita, Noriko Watanabe-Uehara, Kisho Ikeda, Takashi Kitani, Noriyuki Yamashita, Yuhei Kirita, Satoaki Matoba, Benjamin D. Humphreys, Keiichi Tamagaki

CHANGE TO AUTHOR LIST: **Kunihiro Nakai**

Print Name	Signature	Date
**Masahiro Uehara**	Masahiro Uehara	**17th Feb 2020**
**Tetsuro Kusaba**	Tetsuro Kusaba	**17th Feb 2020**
**Tomoharu Ida**	Tomoharu Ida	**17th Feb 2020**
**Kunihiro Nakai**	Kunihiro Nakai	**17th Feb 2020**
**Tomohiro Nakata**	Tomohiro Nakata	**17th Feb 2020**
**Aya Tomita**	Aya Tomita	**17th Feb 2020**
**Noriko Watanabe-Uehara**	上原 [illegible]	**17th Feb 2020**
**Kisho Ikeda**	Kisho Ikeda	**17th Feb 2020**
**Takashi Kitani**	[illegible]	**17th Feb 2020**
**Noriyuki Yamashita**	Noriyuki Yamashita	**17th Feb 2020**
**Yuhei Kirita**	[illegible]	**17th Feb 2020**

**Satoaki Matoba**	Satoaki Matoba	**17th Feb 2020**
**Benjamin D. Humphreys**	Ben Humphreys	**17th Feb 2020**
**Keiichi Tamagaki**	Keiichi Tamagaki	**17th Feb 2020**
